# Flexible Theoretical Calculation of Loop Length and Area Density of Weft-Knitted Structures: Part II

**DOI:** 10.3390/ma14174988

**Published:** 2021-08-31

**Authors:** Edgaras Arbataitis, Daiva Mikucioniene, Tetiana Ielina, Liudmyla Halavska

**Affiliations:** 1 Department of Production Engineering, Kaunas University of Technology, 44249 Kaunas, Lithuania; edgaras.arbataitis@ktu.edu; 2 Department of Textile Technology and Design, Kyiv National University of Technologies and Design, 01011 Kyiv, Ukraine; yelina.tv@knutd.edu.ua (T.I.); galavska.ly@knutd.edu.ua (L.H.)

**Keywords:** loop length, area density, weft-knitted structure, tuck, tuck stitch, geometrical model

## Abstract

A simple and flexible method for theoretical calculation of the main structural parameters of various weft-knitted fancy and combined patterns is presented in this article. It is especially important for patterns containing different elements, such as loops, floats of different lengths, tucks, and tuck stitches. Measurement of an actual average length of the loop in these fabrics is complicated because it is necessary to disassemble precisely one pattern repeat to measure the yarn length and divide it by the number of elements in this pattern repeat. For large and complex pattern repeats, this is difficult and usually gives a high number of errors. It is very important to have lengths of structural elements as it helps to predict the main physical properties of knitted fabrics and their mechanical behaviour, which is especially important for protective textiles. The main idea of the proposed method, based on Čiukas geometrical model, is to calculate lengths of various structural elements or even their parts separately, taking into account the number of needle bars and their formation principle, which gives great flexibility to such modelling. The proposed theoretical formulas can be used for various patterned weft-knitted structures containing not only loops but tucks, floats of different lengths, or additional yarns; they give very few errors in empirical calculations and are easy to use.

## 1. Introduction

The type and length of structural elements of knitted fabrics and their distribution in a pattern repeat are the main factors influencing the physical properties and mechanical behaviour of the knitted fabrics. Knitting elements, such as loops, tucks, floats, additional yarns or their parts, and their distribution in the structure determine the knitting pattern, which is very important for the design of the knit and for many properties of the fabric. Length of the structural elements is another main parameter which determines physical and mechanical properties and enables the comparison of different fabrics knitted in the same pattern [1,2,3,4]. For comparison of basic knitted structures composed only of loops, the loop length can be used as the main factor. However, for structures composed of different structural elements, usually, an average length of the loop in a pattern repeat is used [5]. As the knitted fabrics are increasingly used in technical applications, there is a need for fabrics with different structures and accordingly different physical and mechanical properties, which will consequently behave differently as technical textiles in terms of mechanical properties. Floats of different lengths, tucks, and additional yarns used in the knitted structure enable a great variety of functional properties, such as strength, dimensional stability or exceptional stretch, air and water vapour permeability, etc., which are especially important for products used for technical application [6,7,8,9,10,11]. It is very important to have possibility of an objective prediction of these properties during the designing stage. Geometrical modelling of the knitted structures is also needed to understand the dimensional behaviour of knitted fabrics in order to define the surface properties. However, geometrical modelling is mostly focused on modelling the basic loop geometry. The necessity of the models of a knitted structure including tucks and tuck stitches has been discussed in the literature during the last two decades [12,13]. Nevertheless, only a few works dedicated to this task are published [14,15,16,17,18], and there is still a lack of flexible and simple enough models that could be used for theoretical loop length and especially area density calculations of complex knitted structures.

Based on the findings of a previous study [5], which presents geometrical modelling of basic knitted structures containing only loops, this work is focused on experimentally proving the application of Čiukas geometrical model principle to more complex fancy and combined weft-knitted structures with tucks and tuck stitches in a pattern to show the flexibility of the model. The main advantage of the proposed formulas is that they can be used for the calculation of lengths of knitted elements and the area density of various weft-knitted fabrics.

## 2. Materials and Methods

**Figure 1:**
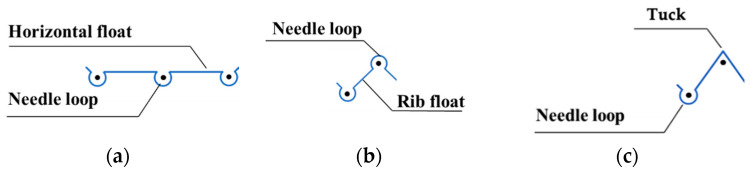
Graphic notation of the needle loop and horizontal float (**a**), rib float (**b**), and tuck (**c**).

A detailed explanation of Čiukas geometrical model is presented in Part I of a previous study [5]. According to the theory, the weft-knitted loop consists of the needle loop (which, in turn, is comprised of the loop head and two legs) and a float, which connects two adjacent needle loops. However, in the case of tuck, there is no connecting float between the tuck and adjacent loops. Graphic notations of structural elements are presented in Figure 1.

As discussed in the previous paper [5], the length of the needle loop has to be calculated by different formulas depending on loop formation, i.e., if the needle loop is formed on a one-needle-bar knitting machine or on a two-needle-bar knitting machine. These formulas are presented in [5].

In further research, while investigating combined patterns, which are formed of the regular loops and floats, and with shorter and/or longer loops and tucks, it was determined that these formulas do not provide accurate enough values of the loop length for the stitches that are higher than the regular stitch. Therefore, new formulas for these loops were introduced. In these formulas, the height of the loop is described with course spacing (*B*), and each loop is marked with an index (*I*), indicating over how many courses the loop is extended. The length of the needle loop formed on a one-needle-bar knitting machine is calculated according to Formula (1) [14], and the length of the needle loop formed on a two-needle-bar knitting machine is calculated according to the Formula (2) [14].

(1) $$l_{si}=0.5\pi \left(0.5A+d\right)+2\frac{I\cdot Q\cdot B}{H};$$

(2) $$l_{di}=0.5\pi \left(0.5A+d\right)+2\sqrt{{\left(\frac{I\cdot Q\cdot B}{H}\right)}^{2}+d^{2}};$$

where *I* is the dimensionless index describing the height of the loop and can be determined according to the numerical matrix of the knitting pattern: 1—for the shortest loop, which extends over one loop course; 2—for the loop which extends over two loop courses; 3—for the loop which extends over three loop courses, etc.; *Q* is the size describing the number of loops in the first wale of the pattern repeat; *H* is the size of the pattern repeat in vertical directions; *A* is the wale spacing, in mm; *B* is the course spacing, in mm; *d* is the yarn diameter, in mm.

For the calculation of the length of the tuck (Figure 1c), two formulas are introduced. The length of the tuck formed on a one-needle-bar knitting machine is calculated according to Formula (3) [14], which better describes tuck that in form is close to an isosceles triangle, and the length of tuck formed on a two-needle-bar knitting machine is calculated according to Formula (4) [14], which describes the tuck that is close in form to an ellipsis.

(3) $$l_{st}=2\sqrt{{\left(n\cdot A\right)}^{2}+{\left(I_{t}\cdot B_{t}\right)}^{2}};\;$$

(4) $$l_{dt}=0.5\pi \left(1.5\left(\frac{A}{N}+I_{t}\cdot B^{\prime }\right)-\sqrt{\frac{A\cdot I_{t}\cdot B^{\prime }}{N}}\right);$$

where *n* is the dimensionless index describing the number of wales having the same tuck; *I_t_* is dimensionless index describing the height of the tuck: 1—for the shortest tuck which extends over one loop course; 2—for the tuck which extends over two loop courses; 3—for the tuck which extends over three loop courses, etc.; *N* is the size describing the number of needle bars; *A* is the wale spacing, in mm; *B_t_* is the height of the tuck, in mm; *B’* is the height of the tuck stitch, in mm; *d* is the yarn diameter, in mm.

Formulas for calculation of the length of the horizontal and rib floats of any lengths are presented in the previous article [5].

In the general case, the area density of a weft-knitted fabric can be calculated by Formula (5) [19,20,21] as follows:

(5) $$M=\frac{LY\cdot T}{A\cdot B\cdot R\cdot H};$$

where *LY* is the total length of the yarn in the pattern repeat, in mm; *T* is linear density of the yarn, in tex; *A* is the wale spacing, in mm; *B* is the course spacing, in mm; *R* and *H* is the size of the pattern repeat in horizontal and vertical directions, respectively.

To find the average value of the loop length $\bar{l}$ in a pattern repeat, the total length of the yarn in the pattern repeat *LY* has to be divided by the number of loops in the pattern repeat.

**Figure 2:**
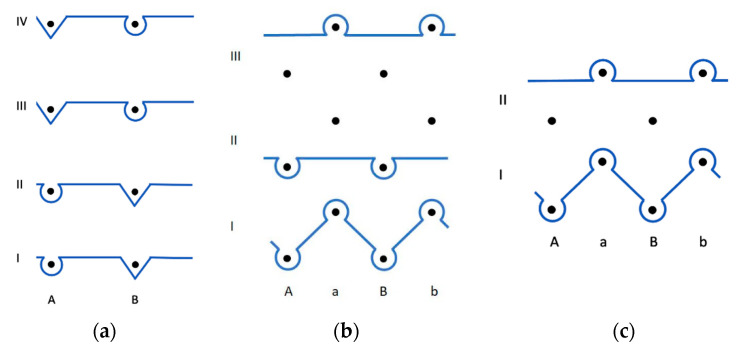

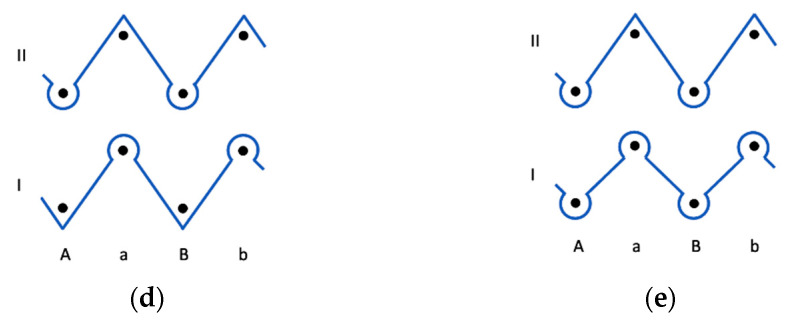
Graphic notation of the investigated knitted structures: (**a**) single tuck stitch; (**b**) Milano rib 1 × 1; (**c**) half-Milano rib 1 × 1; (**d**) cardigan rib 1 × 1; (**e**) half-cardigan rib 1 × 1.

**Figure 3:**
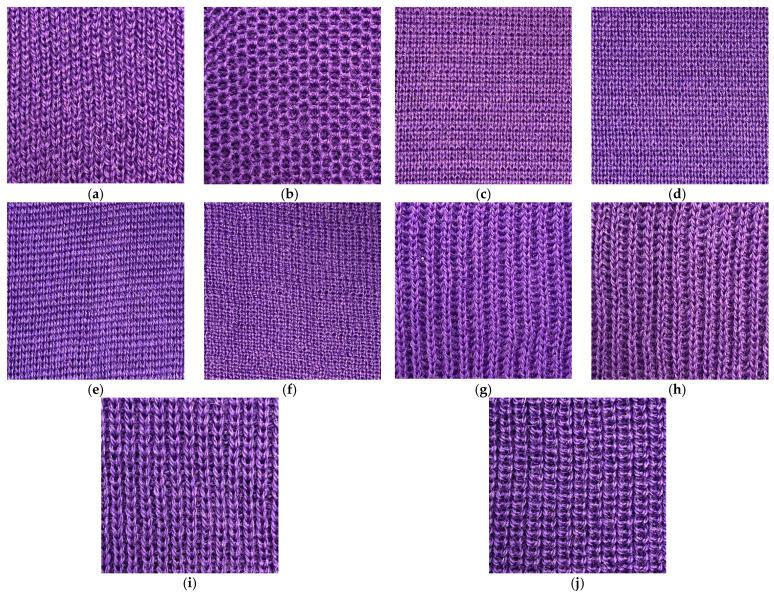
Images of the investigated knitted structures: (**a**) single tuck stitch, technical face side; (**b**) single tuck stitch, technical back side; (**c**) Milano rib 1 × 1, technical face side; (**d**) Milano rib 1 × 1, technical back side; (**e**) half-Milano rib 1 × 1, technical face side; (**f**) half-Milano rib 1 × 1, technical back side; (**g**) cardigan rib 1 × 1, technical face side; (**h**) cardigan rib 1 × 1, technical back side; (**i**) half-cardigan rib 1 × 1, technical face side; (**j**) half-cardigan rib 1 × 1, technical back side.

Experimental samples (presented in Figure 2 and Figure 3) were produced from two-ply blended 50% wool/50% acrylic yarns with linear density 40 tex × 2 on an electronic flat weft knitting machine SES 122 (Shima Seiki, Japan), gauge E12. The samples were knitted from original yarn packages without additional lubrication during knitting.

Structural parameters of the experimental knits were measured according to Standard BS 5441:1998 and are presented in Table 1. All knitted fabrics were investigated in a “grey” state, i.e., after at least 72 h relaxation in a free state in standard atmospheric conditions at 20 ± 2 °C temperature and 65 ± 4% humidity according to Standard EN ISO 139:2005 and without any additional finishing.

## 3. Results and Discussion

### 3.1. Theoretical Calculation of Weft-Knitted Elements Length

Before the application of the model to each pattern, specific structural elements and their number were determined according to the graphical notation of investigated knitted structures (Figure 2). Results are presented in Table 2.

At first glance, Milano rib and half-Milano rib knitting patterns appear to be similar, although Milano rib has one extra course of single jersey loops. Further investigation showed that Milano rib is comprised of two one-needle-bar loops connected with two horizontal floats and two two-needle-bar loops connected with two rib floats, while half-Milano rib is comprised of two two-needle-bar loops, each of them being of different heights, connected with two rib floats and one one-needle-bar loop with adjacent horizontal float.

For the length calculation of loops formed in the second and third courses of Milano rib (see Figure 2b) and in the second course of half-Milano rib (see Figure 2c), the formula of the one-needle-bar loop [5] was used. For Milano rib, course spacing *B* is the same for all the loops, no matter which side of the knit is investigated, as the front face one-needle-bar loops are the same in height as the back face one-needle-bar loops (the same applies to two-needle-bar loops). In the case of half-Milano rib, course spacing is different on both sides of the knit. For the calculation of back-face loop lengths (both one-needle bar and two-needle bar), the front face course spacing (let us call it $B_{F}=1.67$) is divided by 2, since there are two loop courses in the back face ($B_{B}=B_{F}/2=0.835).$ Index *H* is equal, i.e., for Milano rib = 3 and for Half Milano rib = 2. Index Q=2 for Milano rib and Q=2 for Half Milano rib. Both indexes are the same for the one-needle-bar loops and two-needle-bar loops. In both rib cases, index I=1, for one-needle-bar loop. After adding the values of indexes to original formulas for the simplification of the calculations, the following expressions are used for the length calculations of one-needle-bar loop in Milano rib (6) and half-Milano rib (7):

(6) $$l_{MRs1}=0.5\pi \left(0.5A+d\right)+\frac{4B}{3};$$

(7) $$l_{HMRs1}=0.5\pi \left(0.5A+d\right)+B_{B};$$

For the length calculation of loops formed in the first course of both Milano rib and half-Milano rib, the formula of the two-needle-bar loop [5] was used. In the case of Milano rib, two-needle-bar loops are the same in height and index I=1. In the half-Milano rib pattern, two-needle-bar loops are different in height—the front-face loop is extended through two loop courses—index $I=2,\;B_{F}=1.67$ for the front-face loop and I=1, $B_{B}=0.835$ for the back-face loop. The following expressions were used for the calculations of Milano rib (8) and half-Milano rib (I=1) (9); I=2 (10):

(8) $$l_{MRd1}=0.5\pi \left(0.5A+d\right)+2\sqrt{{\left(\frac{2B}{3}\right)}^{2}+d^{2}};$$

(9) $$l_{HMRd1}=0.5\pi \left(0.5A+d\right)+2\sqrt{{\left(\frac{B_{B}}{2}\right)}^{2}+d^{2}};$$

(10) $$l_{HMRd2}=0.5\pi \left(0.5A+d\right)+2\sqrt{{\left(B_{F}\right)}^{2}+d^{2}};$$

In Milano rib and half-Milano rib, the lengths of both horizontal floats and rib floats are calculated separately according to the formulas presented in [5].

In the case of the half-cardigan rib, the pattern (see Figure 2 and Table 1) is comprised of two regular two-needle-bar loops and one two-needle-bar loop that extends over two loop courses—a tuck stitch and a tuck. Similar to half-Milano rib, two different formulas for the loop length calculations have to be used. The formula for the regular two-needle-bar loop length calculation is presented in [5]. For the higher-in-length tuck stitch, Formula (2) was used. According to pattern repeat (Figure 2), Q=2;I=2;H=2. Same as in half-Milano rib, both sides of the fabric have loops of different heights. In this case, the height of the back-face loop, i.e., the tuck loop, is two times greater. Therefore, it is necessary to calculate new course spacing *B’* [14] as follows:

(11) $$B’=B\left(N_{max}+1\right);$$

where *B* is the original course spacing, in mm; *N_max_* is the dimensionless index describing the highest tuck: 1—for the tuck, which extends over one loop course; 2—for the tuck which extends over two loop courses; 3—for the tuck which extends over three loop courses, etc.

In this case, *B*’ is calculated according to Formula (12) and tuck-stitch length is calculated according to Formula (13).

(12) $$B^{\prime }=0.83\left(1+1\right)=1.66\;mm;$$

(13) $$l_{HCRd2}=0.5\pi \left(0.5A+d\right)+2\sqrt{{\left(2B^{\prime }\right)}^{2}+d^{2}};$$

The length of the tuck is calculated according to Formula (14) $I_{t}=1$:

(14) $$l_{CRdt1}=0.5\pi \left(1.5\left(\frac{A}{2}+B^{\prime }\right)-\sqrt{\frac{A\cdot B^{\prime }}{2}}\right);$$

The half-cardigan rib has two rib floats in the first course. In the second course, there are no floats, and the tuck pulls out all the yarn into itself.

In the case of the cardigan rib, the pattern is comprised of two identical, but opposite to each other loop rows. Each of them is comprised of one tuck and one two-needle-bar loop—tuck stitch that extends over two loop rows. Calculations for the cardigan rib are similar as for the half-cardigan rib: for two-needle-bar tuck stitch, Formula (13), and for two-needle-bar tuck, Formula (14) were used (all the dimensionless sizes and indexes are the same for both knitting patterns and $B^{\prime }=1.54$). The only difference in calculations is that for the half-cardigan rib, it is accepted that there are two needle loops in the first wale of the pattern repeat, and for the cardigan rib, we assumed that there are two courses in the pattern, making the vertical repeat of both patterns the same (H=2).

Additionally, there are no horizontal or rib floats because, as mentioned before, tuck is pulling all the yarn into itself.

Tuck-stitch pattern repeat is comprised (see Figure 2 and Table 1) of two regular one-needle-bar needle loops, two one-needle-bar tuck stitches which are stretched over three loop rows, two tucks that are stretched over two loop rows, and two tucks that are the height of one loop row. The regular one-needle-bar needle loop length is calculated according to the one-needle-bar needle-loop length formula presented in [5]. One-needle-bar tuck-stitch length is calculated according to Formula (1), where I=3;Q=2;H=4. The following expression can be used for calculations:

(15) $$l_{si}=0.5\pi \left(0.5A+d\right)+3B^{\prime };$$

where B’ is calculated according to Formula (14), where $N_{max}=2$. We obtain Formula (16) as follows:

(16) $$B^{\prime }=B\left(N_{max}+1\right)=0.65\left(2+1\right)=1.95\;mm;$$

The lengths of the tucks are calculated according to Formula (3), where $n=1;I_{t}=1$ for tuck, which is one row high, and $n=1;I_{t}=2$ for tuck, which is two rows high. The height of each tuck is determined separately—first, tuck extends over one loop row ($B_{t1}=B=0.65\;\mathrm{mm})$, and the second extends over two courses ($B_{t2}=2B=2*0.65=1.30\;\mathrm{mm})$. Taking these changes of the course spacings into the consideration, we obtain the following expressions for the length calculations of tuck one (17) and tuck two (18):

(17) $$l_{st1}=2\sqrt{A^{2}+B_{t1}{}^{2}};$$

(18) $$l_{st2}=2\sqrt{A^{2}+{\left(2B_{t2}\right)}^{2}};$$

There are no horizontal or rib floats.

Results of all the theoretical calculations of the lengths of the structural elements are presented in Table 3.

As all investigated patterns are comprised of different structural elements, which have different shapes and lengths, to calculate the average loop length $\bar{l}$ in a pattern repeat, the total length of the yarn in the pattern repeat *LY* has to be divided by the number of loops in the pattern repeat. The general expression for the total yarn length consumed in a pattern repeat can be expressed by Formula (19) as follows:

(19) $$LY=N_{ls}\cdot l_{s}+N_{ld}\cdot l_{d}+N_{lt}\cdot l_{t}+N_{lh}\cdot l_{h}+N_{lr}\cdot l_{r};$$

where $N_{ls}$ is the number of one-needle-bar needle loops; *l_s_* is the length of one-needle-bar needle loop, in mm; $N_{ld}$ is the number of two-needle-bar needle loops; *l_d_* is the length of two-needle-bar needle loop, in mm; $N_{lt}$ is the number of tucks; *l_t_* is the length of tuck, in mm; $N_{lh}$ is the number of horizontal floats; l_h_ is the length of horizontal float, in mm; $N_{lr}$ is the number of rib floats; *l_r_* is the length of rib float, in mm.

In the case of each knitting pattern, the following expressions were used for the calculation of the total yarn length *LY* in the pattern repeat and the average loop length $\bar{l}$ in the pattern repeat:

(20) $$\mathrm{For}\;\mathrm{Milano}\;\mathrm{rib}:\;LY_{MR}=2l_{MRs1}+2l_{MRd1}+2l_{h}+2l_{r};$$

(21) $${\bar{l}}_{MR}=\frac{LY_{MR}}{N_{Ls1}+N_{Ld1}}=\frac{LY_{MR}}{4};$$

(22) $$\mathrm{For}\;\mathrm{half-Milano}\;\mathrm{Rib}:\;LY_{HMR}=1l_{MRs1}+1l_{MRd1}+1l_{MRd2}+1l_{h}+2l_{r};$$

(23) $${\bar{l}}_{HMR}=\frac{LY_{HMR}}{N_{Ls1}+N_{Ld1}+N_{ld2}}=\frac{LY_{HMR}}{3};$$

(24) $$\mathrm{For}\;\mathrm{cardigan}\;\mathrm{rib}:\;LY_{CR}=2l_{CRd2}+2l_{CRt1};$$

(25) $${\bar{l}}_{CR}=\frac{LY_{CR}}{N_{LCRd2}}=\frac{LY_{CR}}{2};$$

(26) $$\mathrm{For}\;\mathrm{half-cardigan}\;\mathrm{rib}:\;LY_{HCR}=2l_{d}+1l_{HCRd2}+1l_{HCRt1}+2l_{r};.\;$$

(27) $${\bar{l}}_{HCR}=\frac{LY_{HCR}}{N_{ld}+N_{LCRd2}}=\frac{LY_{HCR}}{3};$$

(28) $$\mathrm{For}\;\sin\mathrm{gle}\;\mathrm{tuck}\;\mathrm{stitch}:\;LY_{TS}=2l_{s}+2l_{TSs2}+2l_{TSt1}+2l_{TSt2};$$

(29) $${\bar{l}}_{TS}=\frac{LY_{TS}}{N_{ls}+N_{LTSs2}}=\frac{LY_{TS}}{4};$$

Results of the theoretically calculated total yarn length in the pattern repeat and the average loop length in the pattern repeat for all investigated knits are presented in Table 4.

**Figure 4:**
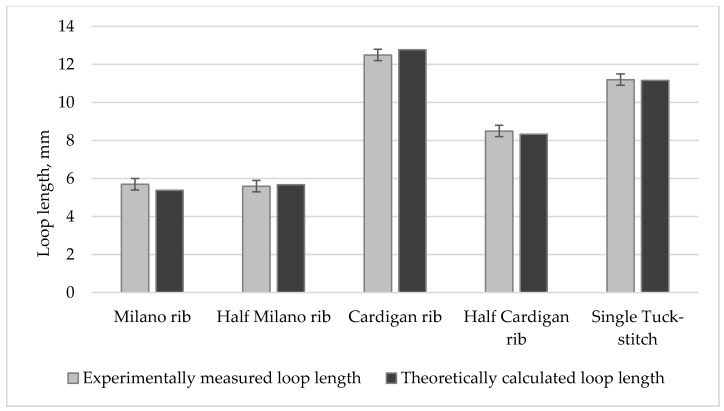
Comparison of theoretically calculated and experimentally measured loop lengths.

A comparison of theoretically calculated loop lengths of investigated knitted structures and their experimentally measured values is presented in Figure 4. For the evaluation of the accuracy of the presented theoretical calculations of the lengths of the structural elements of knits, the relative error values between the theoretically calculated and experimentally measured (see in Table 1) results were calculated. Re-sults presented in Table 4 and Figure 4 clearly demonstrate that the accuracy of the theoretical calculations of the average loop length in comparison to the experimentally measured data is very high in all cases, and in most cases, relative error values did not exceed 5 %. Thus, it is safe to state that the presented model and theoretical formulas for the calculation of the lengths of structural elements can be used for modelling the loop length of various combined and fancy knitted structures containing loops and floats, as well as tucks and tuck stitches, since calculations have very high accuracy. Additionally, it is important that presented Formulas can be applied for very different weft-knitted patterns.

A comparison of theoretically calculated loop lengths of investigated knitted structures and their experimentally measured values is presented in Figure 4. For the evaluation of the accuracy of the presented theoretical calculations of the lengths of the structural elements of knits, the relative error values between the theoretically calculated and experimentally measured (see in Table 1) results were calculated. Results presented in Table 4 and Figure 4 clearly demonstrate that the accuracy of the theoretical calculations of the average loop length in comparison to the experimentally measured data is very high in all cases, and in most cases, relative error values did not exceed 5 %. Thus, it is safe to state that the presented model and theoretical formulas for the calculation of the lengths of structural elements can be used for modelling the loop length of various combined and fancy knitted structures containing loops and floats, as well as tucks and tuck stitches, since calculations have very high accuracy. Additionally, it is important that presented Formulas can be applied for very different weft-knitted patterns.

### 3.2. Theoretical Calculation of Weft-Knitted Fabric Area Density

The accuracy of previous calculations of the loop length was verified by the next step of the application of the geometrical model—theoretical calculation of the area density. For cardigan and half-cardigan ribs, the general Formula (9) was used. In both cases, experimentally measured wale *A* and course *B* spacings were used. The only difference in calculations is that for the half-cardigan rib, it is accepted that there are two needle loops in the first wale of the pattern repeat H=2, and for the cardigan rib, we assumed that there are two courses in the pattern, making the vertical repeat of the pattern same for both knits. In both cases, the horizontal pattern repeat is R=2.

For the calculation of area density of Milano and half-Milano ribs, an alteration to the original Formula (9) had to be made, as we must take into account the number of needle bars. The following Formula (30) was used for the calculations of area density:

(30) $$M=\frac{LY\cdot T\cdot N}{A\cdot B\cdot R\cdot H};$$

where *LY* is the total length of the yarn in the pattern repeat (in mm), which is the sum of the lengths of all elements, i.e., all needle loops and floats in the pattern repeat; *T* is linear density of the yarn, in tex; *N* is dimensionless size describing the number of needle bars; *A* is the wale spacing, in mm; *B* is the course spacing, in mm; *R* and *H* is the size of the pattern repeat in horizontal and vertical directions, respectively.

Vertical and horizontal repeats for Milano rib are H=2, R=2 and for half-Milano rib, respectively, are H=1, R=2.

For the calculation of area density of single tuck-stitch knit, a different alteration to general Formula (9) was made. An average loop height (course spacing) $\bar{B}$ was calculated (31), considering only the size of two loops, i.e., the regular one-needle-bar loop and the tuck stitch. Formula (32) was used to calculate the area density of the single tuck-stitch knit:

(31) $$\bar{B}=\frac{B+B’}{2}=\frac{0.65+1.95}{2}=1.30\;\mathrm{mm};$$

(32) $$M=\frac{LY\cdot T}{A\cdot \bar{B}\cdot R\cdot H};$$

where vertical and horizontal repeats are H=2, R=2, respectively.

Results of the area density theoretical calculations are presented in Table 5.

**Figure 5:**
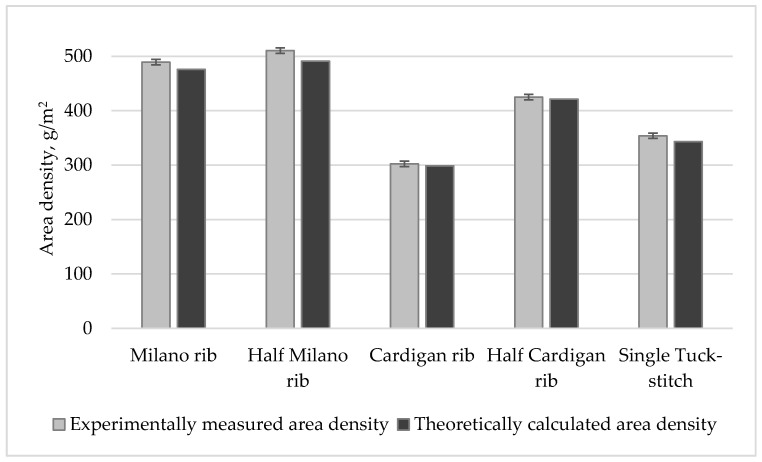
Comparison of theoretically calculated and experimentally measured area densities.

A comparison of theoretically calculated area density of investigated knitted structures and their experimentally measured values is presented in Figure 5. The presented results clearly demonstrate that theoretical and experimental values of the area density are very close for all investigated weft-knitted structures. The relative error between the theoretically calculated and experimentally measured area density values (presented in Table 5) indicates very high accuracy of the presented formula and its modifications for area density calculation of each pattern, as the relative error values are very low, in most cases lower than 3%, thus proving that proposed geometrical model and theoretical formulas can be recommended for various combined structures.

## 4. Conclusions

The presented theoretical calculations demonstrate that mathematical formulas and their alterations for the theoretical calculations of the loop length and area density, developed according to Čiukas geometrical model, give very high accuracy for various fancy and combined structured knits, containing in the pattern repeat various structural elements, such as loops, floats, tucks, and tuck stitches. The relative error of theoretical calculation of the loop length of various knits with different pattern repeats did not exceed 6% and in most cases was lower than 2.5%. The relative error of theoretical calculation of the area density of investigated knits in most cases was lower than 3%. Although the calculations and the application of the geometrical model to fancy and combined weft-knitted structures are becoming more complex, they are still relatively simple and easy to use. The main advantage of the proposed formulas is that they can be applied to various weft-knitted structures, containing different sized loops, tucks, and floats, irrespective of pattern complexity or size of pattern repeats.

## Figures and Tables

**Table 1:** Structural parameters of the investigated knits.

Pattern	Yarn Diameter *d*, mm	Wale Density *P_w_*, cm^−1^	Course Density *P_c_*, cm^−1^	Wale Spacing *A*, mm	Course Spacing *B*, mm	Loop Length *l*, mm	Area Density *M*, g/m^2^
Milano rib	0.4	6.5 ± 0.2	8.5 ± 0.2	1.54	1.18	5.7 ± 0.2	489.2 ± 5
Half-Milano rib		6.0 ± 0.2	6.0 ± 0.1	1.67	1.67	5.6 ± 0.2	510.3 ± 5
Cardigan rib		4.5 ± 0.2	13.0 ± 0.3	2.22	0.77	12.5 ± 0.3	302.4 ± 4
Half-cardigan rib		7.0 ± 0.2	12.0 ± 0.2	1.43	0.83	8.5 ± 0.2	424.8 ± 5
Single tuck stitch		5.0 ± 0.2	15.5 ± 0.3	2.00	0.65	11.2 ± 0.3	353.9 ± 4

**Table 2:** Number of structural elements in pattern repeat.

Pattern	Number of One-Needle-Bar Needle Loops *N_ls_*		Number of Two-Needle-Bar Needle Loops *N_ld_*		Number of One-Needle-Bar Tucks Loops *N_lst_*		Number of Two-Needle-Bar Tucks *N_ldt_*	Number of Horizontal Floats *N_lh_*	Number of Rib Floats *N_lr_*
Loop/tuck/float index	*I* = 1	*I* = 3	*I* = 1	*I* = 2	*I* = 1	*I* = 2	*I* = 1	*I* = 2	*I* = 1
Milano rib	2	-	2	-	-	-	-	2	2
Half-Milano rib	1	-	1	1	-	-	-	1	2
Cardigan rib	-	-	-	2	-	-	2	-	-
Half-cardigan rib	-	-	2	1	-	-	1	-	2
Single tuck stitch	2	2	-	-	2	2	-	-	-

**Table 3:** Theoretically calculated lengths of structure elements.

Pattern	One-Bar Needle Loop *l_s_*, mm		Two-Bars Needle Loop *l_d_*, mm		One-Bar Tuck *l_st_*, mm		Two-Bar Tuck *l_dt_*, mm	Horizontal Float *l_h_*, mm	Rib Float *l_r_*, mm
Loop/tuck/float index	*I* = 1	*I* = 3	*I* = 1	*I* = 2	*I* = 1	*I* = 2	*I* = 1	*I* = 2	*I* = 1
Milano rib	3.4	-	3.6	-	-	-	-	1.84	1.93
Half-Milano rib	2.77	-	3.09	5.36	-	-	-	1.94	1.94
Cardigan rib	-	-	-	8.58	-	-	4.19	-	-
Half-cardigan rib	-	-	4.41	8.46	-	-	3.89	-	1.92
Single tuck stitch	3.49	8.00	-	-	4.20	6.53	-	-	-

**Table 4:** Theoretically calculated yarn length in pattern repeats, average loop length, and relative error between the theoretically calculated and experimentally measured values.

Pattern	Yarn Length in the Pattern Repeat *LY*, mm	Average Loop Length in the Pattern Repeat $\bar{l}$, mm	Relative Error between Theoretical and Experimental Values, %
Milano rib	21.53	5.38	5.58
Half-Milano rib	17.04	5.68	2.42
Cardigan rib	25.53	12.77	2.12
Half-cardigan rib	24.98	8.33	2.06
Single tuck stitch	44.62	11.16	0.39

**Table 5:** Area density and relative error between the theoretically calculated and experimentally measured values.

Pattern	Theoretically Calculated Area Density *M,* g/m^2^	Relative Error between Theoretical and Experimental Values, %
Milano rib	475.77	2.75
Half-Milano rib	490.88	3.80
Cardigan rib	298.70	1.23
Half-cardigan rib	421.27	0.82
Single tuck stitch	343.27	3.00

## Data Availability

Not applicable.
